# Simultaneous Live Cell Imaging Using Dual FRET Sensors with a Single Excitation Light

**DOI:** 10.1371/journal.pone.0006036

**Published:** 2009-06-24

**Authors:** Yusuke Niino, Kohji Hotta, Kotaro Oka

**Affiliations:** Center for Biosciences and Informatics, School of Fundamental Science and Technology, Keio University, Kohoku-ku, Yokohama, Japan; University of Oldenburg, Germany

## Abstract

Fluorescence resonance energy transfer (FRET) between fluorescent proteins is a powerful tool for visualization of signal transduction in living cells, and recently, some strategies for imaging of dual FRET pairs in a single cell have been reported. However, these necessitate alteration of excitation light between two different wavelengths to avoid the spectral overlap, resulting in sequential detection with a lag time. Thus, to follow fast signal dynamics or signal changes in highly motile cells, a single-excitation dual-FRET method should be required. Here we reported this by using four-color imaging with a single excitation light and subsequent linear unmixing to distinguish fluorescent proteins. We constructed new FRET sensors with Sapphire/RFP to combine with CFP/YFP, and accomplished simultaneous imaging of cAMP and cGMP in single cells. We confirmed that signal amplitude of our dual FRET measurement is comparable to of conventional single FRET measurement. Finally, we demonstrated to monitor both intracellular Ca^2+^ and cAMP in highly motile cardiac myocytes. To cancel out artifacts caused by the movement of the cell, this method expands the applicability of the combined use of dual FRET sensors for cell samples with high motility.

## Introduction

Many genetically encoded sensors have been developed based on fluorescence resonance energy transfer (FRET), the radiationless transfer of excited state energy from an excited donor to an acceptor, between fluorescent proteins [1]–[3]. These biosensors provide a means to image the spatiotemporal dynamics of various intracellular signals including second messengers, protein-protein interactions and enzyme activities. Moreover, combined use of the sensors would be useful to correlate multiple signaling events for understanding complex signal transduction networks. However, to date, most FRET applications have used only a single sensor in a cell. The sensor has a broad spectral profile for two fluorescent proteins, hence, when the several sensors are present at the same location, imaging without the significant spectral overlap is difficult.

Recently, strategies to overcome this problem, using FRET pairs of ECFP/EYFP and mOrange/mCherry [4], mTFP1/mCitrine and mAmetrine/tdTomato [5] and ECFP/Venus and TagRFP/mPlum [6], have been reported. These dual FRET pairs are spectrally compatible, when the donors are excited alternately at two different wavelengths and the emissions are collected sequentially. But when used simultaneous excitation not sequential excitation for two donors, sensitized emission from the first acceptor YFP should still be detected in the second donor fluorescence channel, resulting in possible artifacts. Since alteration of excitation light between two different wavelengths necessitates a lag time, sequential acquisition in the previous strategies is not adequate to follow fast signal dynamics or signal changes in highly motile cells.

Here we report a method for imaging of two FRET pairs excited simultaneously with a single excitation light. We constructed FRET sensors using Sapphire/RFP for combined use with CFP/YFP, and both donors were excited with a violet light. We detected the emissions from the two FRET pairs using a quad channel imager without a lag time, and then distinguished between four fluorescent proteins using a computational method, linear unmixing, that has been recently used to extract the individual contributions of fluorophores which are linearly summated on the spectral detection channels [7]–[9].

First, we tried to image intracellular cAMP and cGMP in single cells, since various FRET sensors for these cyclic nucleotides have been developed [10]–[16] but there is no report on simultaneous measurement of them. The result ensured that our dual FRET approach provides efficient detection which is comparable to conventional single FRET experiments. Next, we demonstrated to monitor both intracellular cAMP and Ca^2+^ in single cardiac myocytes showing periodic contraction. Our method enabled ratiometric measurements for two sensors to cancel out artifacts caused by contracting movement of the cell. Thus, we proposed an alternative approach for imaging of dual FRET sensors in a single cell, more suitable for highly motile cell samples.

## Results

### Setup for dual FRET measurement with a single excitation light

**Figure 1:**
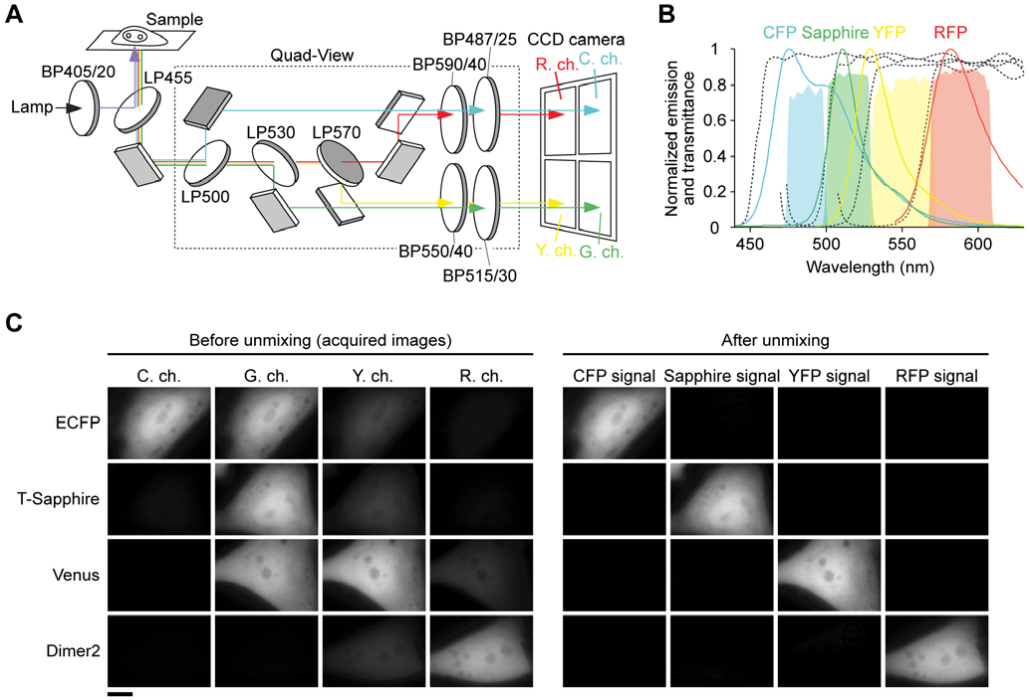
Microscope for simultaneous imaging of dual FRET pairs. (A) Schematic representation of an optical setup with single excitation and four-color channel detection. LP, long pass dicroic mirror. BP, band pass excitation or emission filter. Numbers after LP and BP, wavelength and wavelength/half-bandwidth (nm), respectively. (B) Emission spectra of fluorescent proteins and transmittances of dichroic mirrors and emission filters. Broken lines from left to right indicate the transmission curves of LP455, 500, 530, and 570, and cyan, green, yellow, and red shaded areas represent of BP487/25, 515/30, 550/40, and 590/40 in (A). (C) Acquired images in each detection channel (left) and the images after linear unmixing (right) of HeLa cells expressing each fluorescent protein. Scale bar, 10 µm.

For simultaneous imaging of two FRET sensors based on CFP/YFP and Sapphire/RFP, we excited both donors using a violet light (405 nm) with little direct excitation of YFP and RFP acceptors, and acquired four-color images by placing a quad channel imager (Quad-View, Optical Insights) in front of a CCD camera (Figure 1A), because the number of detection channels that equals the number of fluorophores is required to apply linear unmixing approach [8]. The quad channel imager contains three dichroic mirrors and four emission filters, and the split fluorescence images are projected on the camera with adjustable mirrors. We built up it to collect emissions at 487, 515, 550, and 590 nm for cyan (C.), green (G.), yellow (Y.), and red detection channels (R. ch.s), respectively (Figure 1B).

To determine the relative contribution of each fluorescent protein to each detection channel as reference, we acquired the four-color images of HeLa cells expressing individual fluorescent proteins, ECFP, T-Sapphire [17], Venus [18], and dimer2 RFP variant [19]. Using this reference, then indeed we confirmed that our method based on simultaneous four-color imaging and subsequent linear unmixing is sufficient to distinguish these fluorescent proteins expressed in the cell (Figure 1C).

A caveat to using a quad channel imager for a single CCD camera is that the acquired images on this setup contained geometrical distortion (Figure S1A) and uneven intensity distribution (Figure S1B), that were not eliminated by even a thorough alignment of the adjusting mirrors in the imager. This spatial mismatch might cause significant artifact in the spectral unmixing, therefore, to minimize it, we preprocessed a geometrical correction of the distorted images using projective transformation [20] (Figure S1C-E) and a correction of uneven intensity distribution using reference images of a dye mixture (Figure S1F) for all dual FRET experiments.

### Simultaneous imaging of cAMP and cGMP

**Figure 2:**
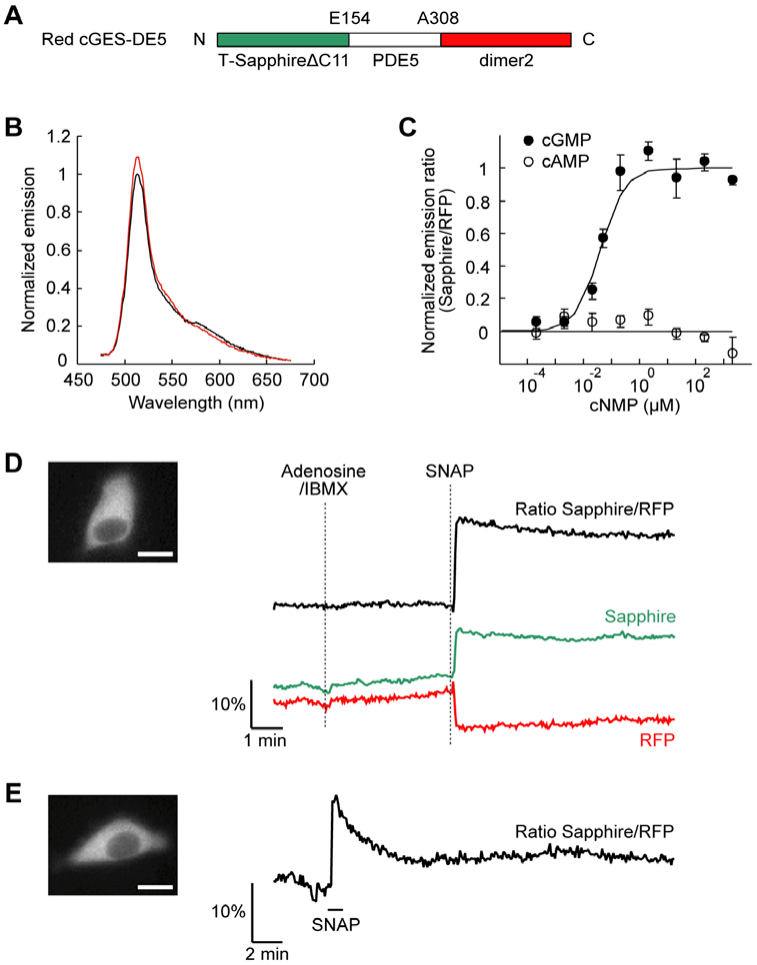
cGMP sensor using FRET with Sapphire/RFP red cGES-DE5. (A) Domain structure of red cGES-DE5. (B) *In vitro* emission spectra of red cGES-DE5 expressed and isolated from HEK293T cells. Black and red lines represent the spectra at zero and saturated cGMP (200 µM), respectively. (C) Concentration response curves of red cGES-DE5 for cGMP and cAMP. Curves were determined *in vitro* from the change in emission ratio at 510 nm (Sapphire) to 580 nm (RFP) (*n* = 4). Half-maximal effective concentration (EC_50_) value for cGMP was 40±8 nM (means±s.e.m.). (D) cGMP imaging by using red cGES-DE5 in PC12 cells. Representative fluorescence image in G. ch. (left) and traces (right) are shown. Typical response to stimulation with 5 µM adenosine and 100 µM IBMX for cAMP and subsequent stimulation with 2 µM SNAP for cGMP (*n* = 9). (E) SNAP washout experiments in PC12 cells expressing red cGES-DE5. Representative fluorescence image in G. ch. (left) and time trace of the FRET signal when stimulated with 5 µM SNAP (right) are shown (*n* = 8). Applied SNAP was washed out by continuous superfusion within 1 min. Scale bar, 10 µm.

cAMP and cGMP are important second messengers, and signaling crosstalk between them is involved in several cell functions [21]–[23]. These cyclic nucleotides have been recently visualized by FRET sensors [10]–[16], but the simultaneous imaging in single cells has never been reported. To test the applicability of our strategy for dual FRET measurement, we first constructed a novel cGMP sensor using Sapphire/RFP and attempted the combind use with a cAMP sensor using CFP/YFP, Epac1-camps [11]. Similarly to a previously described cGMP sensor cGES-DE5 [16], we sandwiched a cGMP binding domain from a phosphodiesterase (PDE5) between T-Sapphire and dimer2, and named the sensor red cGES-DE5 (Figure 2A). We investigated cGMP affinity and selectivity of the sensor using isolated proteins from transiently transfected HEK293T cells (Figure 2B and C). Upon addition of cGMP, in contrast to cGES-DE5, red cGES-DE5 exhibited a decrease in FRET (Figure 2B). Although the FRET change in magnitude is modest relative to of the previous reported sensors [14]–[16], this sensor had both of high cGMP affinity (40 nM) and high selectivity for cGMP over cAMP (>1000-fold) (Figure 2C).

**Figure 3:**
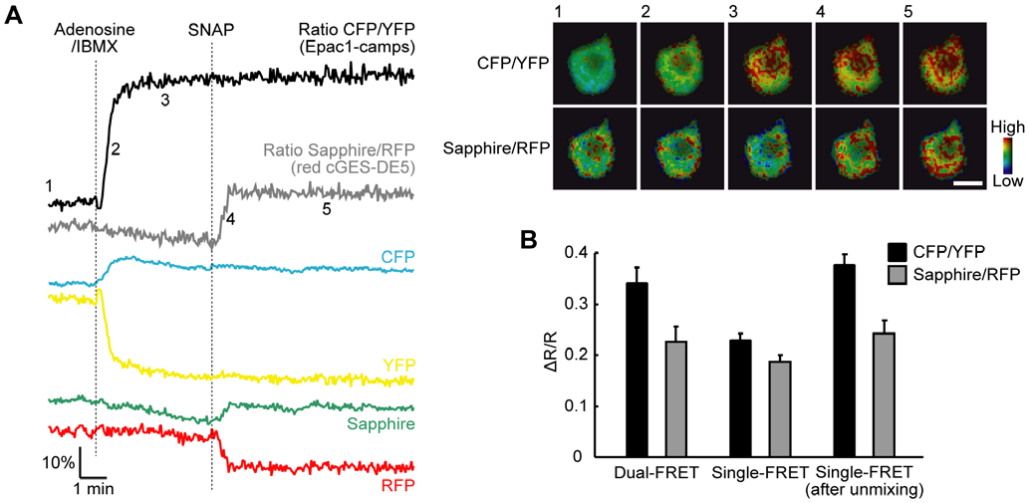
Simultaneous imaging of cAMP and cGMP using dual FRET sensors. (A) Representative traces (left) and pseudocolored ratio images at the indicated time points (right) of Epac1-camps and red cGES-DE5 coexpressed in PC12 cells. The cells were first stimulated with 5 µM adenosine and 100 µM IBMX and subsequently with 2 µM SNAP (*n* = 10). Scale bar, 10 µm. (B) Quantitative analysis of FRET responses of Epac1-camps (CFP/YFP) and red cGES-DE5 (Sapphire/RFP) for adenosine/IBMX and SNAP, respectively. Responses in dual FRET measurement (left), single FRET measurement without any bleedthrough correction (center, C. ch./Y. ch. and G. ch./R. ch.) and that with linear unmixing (right) are compared. Values are means±s.e.m. from at least nine cells.

To examine the ability as a cGMP selective sensor within living mammalian cells, we transfected rat pheochromocytoma PC12 cells, which express endogenous adenosine A_2A_ receptors and the cAMP response to adenosine is known [24]. We stimulated the cells transiently expressing red cGES-DE5 first with adenosine and isobutylmethylxanthine (IBMX) to induce intracellular cAMP increase and then with a NO donor *S*-nitroso-*N*-acetylpenicillamine (SNAP) for cGMP production (Figure 2D). While adenosine/IBMX-induced cAMP increase was detectable by using the cAMP sensor Epac1-camps (Figure S2), red cGES-DE5 did not react to the adenosine/IBMX stimulation and responded with a decrease in FRET for SNAP (Figure 2D), confirming that the sensor shows the cGMP selective response with sufficient amplitude (∼19%, Figure 3B) to detect in living cells. Additionally, to investigate the reversibility of red cGES-DE5, we observed the response when the applied SNAP was washed out by continuous superfusion (Figure 2E). Red cGES-DE5 showed a transient FRET response for a short pulse stimulation of SNAP, confirming that the reversibility of the sensor is preserved despite its high cGMP affinity.

Then, we tried dual FRET experiments in the cells coexpressing Epac1-camps and red cGES-DE5 (Figure 3A). With linear unmixing, adenosine/IBMX stimulation increased only the emission ratio of CFP/YFP for cAMP, whereas the ratio of Sapphire/RFP for cGMP did not change. By subsequent application of SNAP, the Sapphire/RFP ratio underwent an increase but the CFP/YFP ratio was unchanged. These results were consistent with the observations in the cells expressing each FRET sensor (Figure 2D and S2), indicating that our method sufficiently deconvolves the contributions of fluorescence signals made by each sensor. Meanwhile, in the case without linear unmixing (Figure S3), adenosine/IBMX-induced cAMP production increased even the ratio for cGMP (G. ch./R. ch.) together with the ratio for cAMP (C. ch./Y. ch.). This artifact clarifies the need of linear unmixing to discriminate between two FRET signals in our method.

Comparing between dual and single FRET experiments, we found that response amplitude in dual FRET measurement is larger than in single FRET measurement using each sensor without any bleedthrough correction (Figure 3B). This improvement of response amplitude is corresponding to a recent report using multiple channel detection and spectral unmixing for a single FRET sensor [25]. We also confirmed that linear unmixing expands the amplitude in single FRET experiments to a comparable level with dual FRET results (Figure 3B). This means that linear unmixing permits us to compare and analyze the response amplitude of this dual FRET measurement with of conventional single FRET measurement.

### Simultaneous imaging of cAMP and Ca^2+^ in highly motile cardiac myocytes

A primary advantage of a single-excitation dual-emission ratiometric sensor is that its readout is not affected by sample movement in addition to uneven distribution of the sensor and variations in cell thickness. For example of cAMP imaging in neonatal rat cardiac myocytes expressing Epac1-camps (Figure S4), spontaneous contraction of the cell resulted in periodic spikes of CFP and YFP fluorescence intensities, but the emission ratio of CFP/YFP canceled out this artifact caused by the cell movement and correctly reported cAMP production induced by isoproterenol. Finally, we demonstrated to apply our single-excitation dual-FRET method to simultaneous imaging of cAMP and Ca^2+^ in these highly motile cells.

**Figure 4:**
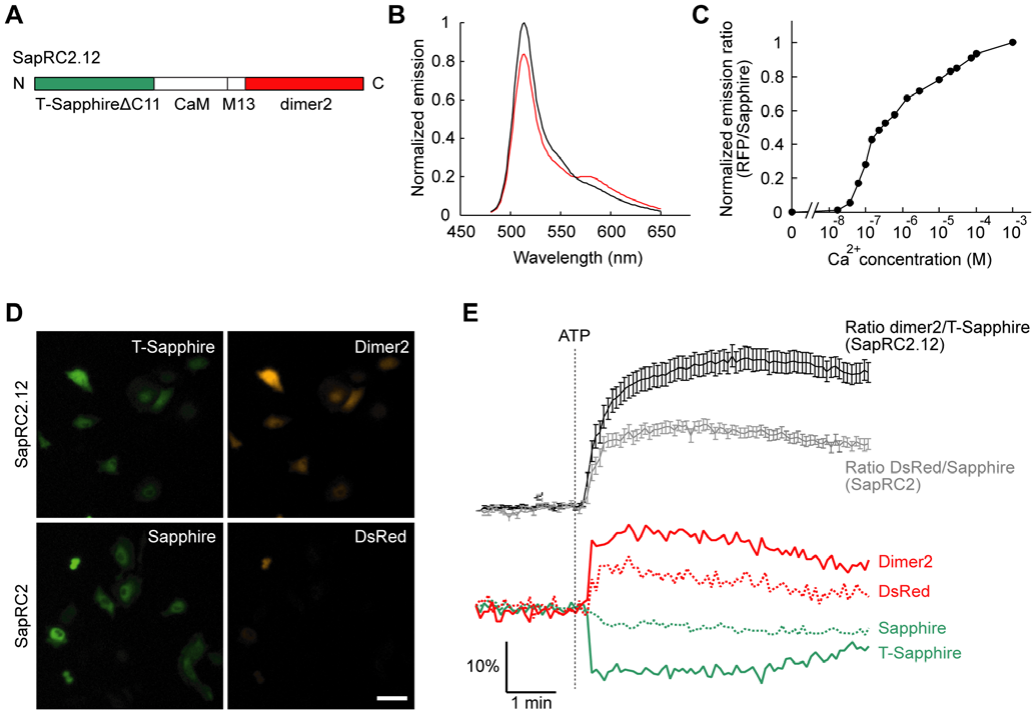
Ca^2+^ sensor using FRET with Sapphire/RFP SapRC2.12. (A) Domain structure of SapRC2.12. (B) *In vitro* emission spectra of SapRC2.12 at zero (black) and saturated Ca^2+^ (2 mM, red). (C) Ca^2+^ titration curve of SapRC2.12. (D) Comparison of fluorescence in HeLa cells expressing SapRC2.12 and SapRC2. Sapphires and RFPs were excited at 405 nm and 550 nm and detected at 535 nm and 605 nm, respectively. Scale bar, 100 µm. (E) Comparative FRET measurements of Ca^2+^ in HeLa cells expressing SapRC2.12 and SapRC2. ATP (100 µM) responses reported by each sensor (upper, means±s.e.m., *n* = 30) and representative traces of fluorescence intensities in a cell were shown (lower).

To use with Epac1-camps, we constructed a Ca^2+^ indicator by fusion of T-Sapphire, calmodulin (CaM), M13 fragment from myosin light chain kinase, and dimer2, and named it SapRC2.12 (Figure 4A), as a successor to a previous reported red cameleon using Sapphire and wild-type DsRed, SapRC2 [26]. *In vitro* characterization (Figure 4B and C), SapRC2.12 exhibited high affinity for Ca^2+^ (apparent Kd, 270 nM) comparable to of SapRC2 [26]. To compare the ability of these two red cameleons expressed in living cells, we transfected HeLa cells with the same amount of cDNAs encoding either SapRC2.12 or SapRC2. One day after transfection, the both sensors expressed in the cells produced bright green fluorescence, but red fluorescence of dimer2 in SapRC2.12 was clearly brighter than of wild-type DsRed in SapRC2 (Figure 4D). And, upon application of ATP, SapRC2.12 gave ∼1.8-fold larger responses than SapRC2 (Figure 4E), confirming improvement of the sensor by the fluorescent proteins with fast maturation [17], [19].

**Figure 5:**
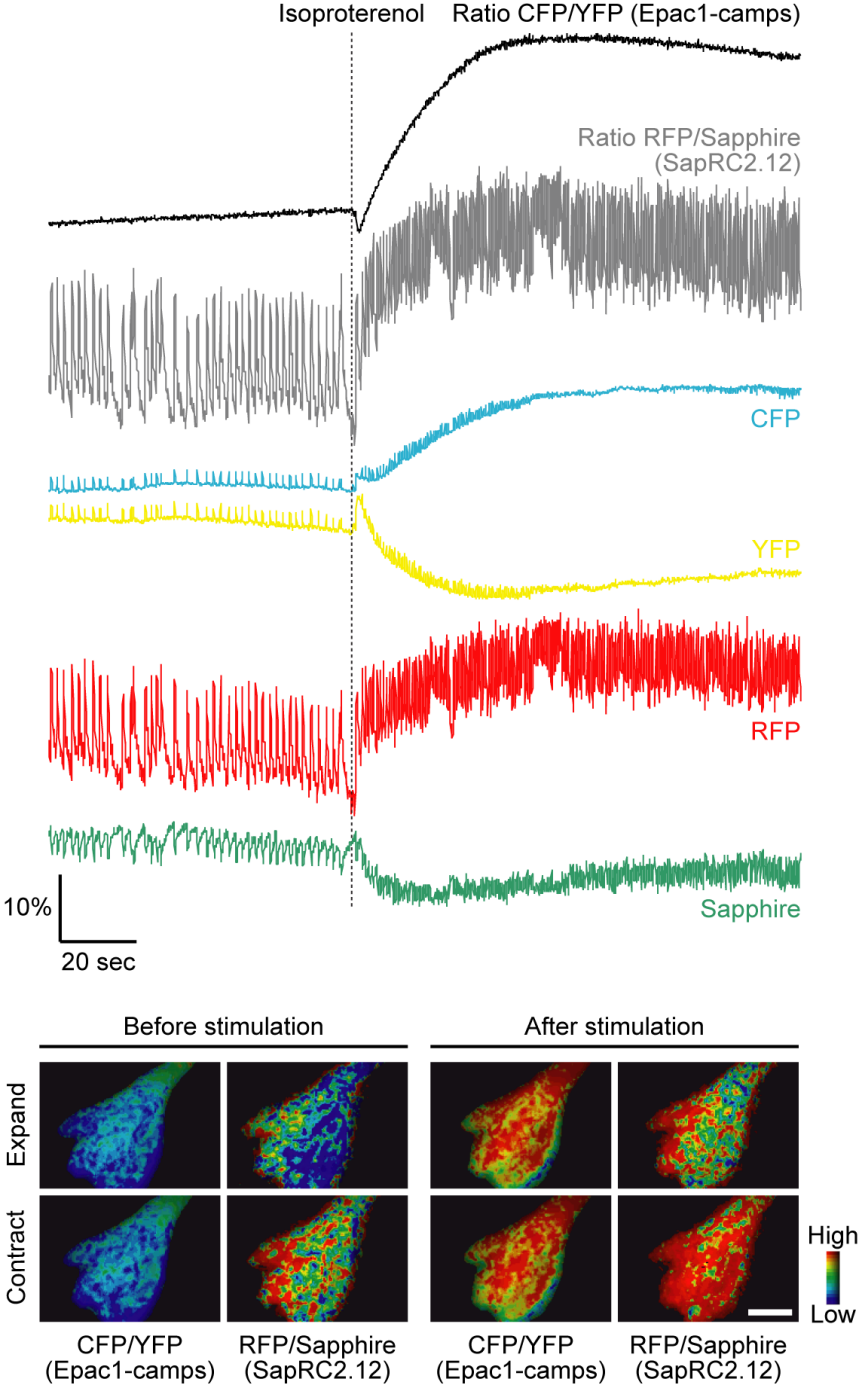
Simultaneous imaging of cAMP and Ca^2+^ in spontaneously contracting cardiac myocytes. Representative traces (upper) and pseudocolored ratio images (lower) of Epac1-camps and SapRC2.12 coexpressed in the cells stimulated with 10 µM isoproterenol are shown (*n* = 9). Scale bar, 10 µm.

We then cotransfected the cardiac myocytes with Epac1-camps and SapRC2.12 and tried dual FRET measurement of cAMP and Ca^2+^ in spontaneous contraction of the cell (Figure 5). After linear unmixing, the ratio of RFP/Sapphire showed periodic spikes (0.38±0.06 Hz, *n* = 9), indicating contraction-coupled increase of cytosolic Ca^2+^, and the ratio of CFP/YFP for cAMP did not fluctuate. Application of isoproterenol led to increases of both FRET signals, and of the Ca^2+^ oscillation frequency (1.43±0.14 Hz). This frequency increase of the Ca^2+^ oscillation (increase rate, 4.53±0.97) consists with the previous study which reported Epac activation triggers it [27]. Again, before linear unmixing the ratio for cAMP (C. ch./Y. ch.) was fluctuated by the cell contraction (Figure S5), showing that combination of spectral imaging and subsequent linear unmixing is necessary to eliminate this artifact and monitor the cAMP change correctly as well as in the cell expressing a single sensor (Figure S4).

## Discussion

In previous reported strategies for imaging of two FRET pairs [4]–[6], to avoid the fluorescence leakage from the first FRET acceptor into the second donor channel, the donors are excited at two different wavelengths, and the resultant fluorescence intensities should be measured sequentially. This procedure causes a lag time for image acquisition, and therefore is inadequate for following fast signal dynamics or signal changes in highly motile cells. To overcome this limitation, we combined an optical setup for simultaneous four-color imaging and a post-process of linear unmixing, and eventually achieved dual FRET measurement of cAMP and Ca^2+^ in contracting movement of the cardiac myocytes (Figure 5). For example of ratiometric imaging of cAMP and Ca^2+^ in single cells, an approach using combination of Epac1-camps and dual-excitation ratiometric Ca^2+^ indicator Fura-2 [28] has been reported. But this also requires filter changing or laser switching and thus would be unsuitable for high-speed simultaneous imaging as described above.

For combining with sensors based on FRET between CFP and YFP, we reported novel sensors using a pair of Sapphire and RFP. In a previous investigation of cGMP sensors with CFP/YFP [16], CGY sensor [14] had very high cGMP affinity (∼20 nM) but low cGMP selectivity (<tenfold), and cygnet [15] and cGES-DE5 sensors [16] had high cGMP selectivity (∼400–600-fold) but affinities in the low-micromoler range (∼1.5–1.7 µM). In that respect, red cGES-DE5 is a unique sensor, which had both of high cGMP affinity (40 nM) and selectivity (>1000-fold) (Figure 2C). We also showed the advantage of the Ca^2+^ sensor SapRC2.12 over the precursor SapRC2 [26] (Figure 4D and E). Thus, these T-Sapphire/dimer2 sensors have some preferable properties and sufficient ability to monitor in living cells (Figure 2D and 4E), however, *in vitro* characterization showed that the FRET response is slightly small (Figure 2B and 4B). During the course of this study, bright red fluorescent proteins, such as tdTomato [29] and TagRFP [30], have been reported. These superior acceptors and circular permutation of fluorescent proteins [31]–[33] might expand the magnitude of FRET response and improve the signal-to-noise ratio.

At the time of writing, a study reported that a combination of four channel detection and spectral unmixing improves FRET signal amplitude of a single Ca^2+^ sensor [25]. In the experiments for cAMP and cGMP imaging, we also showed that linear unmixing improved response amplitude in single and dual FRET measurements (Figure 3B). In our results, this improvement in Epac1-camps (∼1.65-fold) was larger than in red cGES-DE5 (∼1.3-fold) (Figure 3B). A reason for this would be that emissions of CFP and YFP have a broader spectral overlap than of Sapphire and RFP (Figure 1B), resulting in the different effect of linear unmixing.

In conclusion, we established a method for ratiometric imaging of dual FRET pairs simultaneously excited with a single wavelength light, and thereby broadened the employability of the multiparameter fluorescence imaging to a large variety of cell samples including highly motile cells. Aided by this method and a number of combinations of FRET sensors, monitoring multiple biological parameters in individual cells would accelerate to analyze complex signal transduction networks.

## Methods

### Molecular cloning

To construct red cGES-DE5, a fragment of human PDE5A1 (amino acids 154–308) was N-terminally fused to T-Sapphire [17] whose C-terminus was truncated by eleven amino acids (T-SapphireΔC11) using a tripeptide (Arg-Met-His) containing a *Sph*I restriction site, and was C-terminally fused to dimer2 [19] using a dipeptide (Glu-Leu) encoded by a *Sac*I site. To make SapRC2.12, a fragment of CaM-M13 with 5′ *Sph*I and 3′ *Sac*I sites from YC2.60 [31] was sandwiched between T-SapphireΔC11 and dimer2. For mammalian expression, these were subcloned into the *Hind*III/*Eco*RI sites of pcDNA3.1(+) vector (Invitrogen) with a Kozak consensus sequence at the 5′ end. For *in vitro* characterization, SapRC2.12 was subcloned into the bacterial expression vector pRSET_B_ (Invitrogen) at the *Bam*HI/*Eco*RI sites.

pECFP-C1 (Clontech), Venus/pCS2 [18], T-SapphireΔC11 [17], and dimer2 [19] subcloned into the pcDNA3.1(+) vector, Epac1-camps/pcDNA3 [11] and SapRC2/pcDNA3 [26] were used for expression of single fluorescent proteins and FRET sensors.

### Protein expression and *in vitro* spectroscopy

Red cGES-DE5 was transiently transfected into HEK293T cells using Fugene6 (Roche), according to the manufacturer's instructions. After 24 h transfection, cells were washed three times with chilled PBS, scraped from the plate, and resuspended in 5 mM Tris-HCl, 2 mM EDTA (pH = 7.3). Following lysis by sonication (1 pulse) for 5 s on ice, cytosol was obtained by centrifugation at 100,000 *g* for 30 min at 4°C, and analyzed with cGMP and cAMP (Sigma).

SapRC2.12 with an N-terminal polyhistidine tag was expressed in *E. coli* BL21 (DE3) strain (Invitrogen). Cultures were grown at 37°C to an optical density of 0.4–0.5 at 600 nm, and then protein production was induced with 1 mM isopropyl-β-D-thiogalactoside (IPTG) overnight at room temperature. Pellets were lysed by freezing (for 30 min at −20°C) and thawing (for 30 min at room temperature) three times in a solution of 25 mM Tris-HCl (pH 8.0), 1 mM β-mercaptoethanol and a protease inhibitor cocktail for use with bacterial cells (Sigma). Protein purification was carried out using His GraviTrap (GE Healthcare). Purified proteins were dialyzed into 100 mM KCl and 30 mM MOPS, pH 7.2 using a PD-10 column (GE Healthcare). Ca^2+^ titration was done with premixed Ca^2+^ buffers (Calcium Calibration Buffer Kit #2 and #3, Invitrogen).

We measured concentration response curves of the sensors with a fluorescence spectrometer SpectraMax Gemini XS (Molecular Devices).

### Cell imaging

HeLa cells, PC12 cells and rat neonatal cardiac myocytes were grown on a 35-mm glass bottom dish (Iwaki) and transiently transfected with cDNAs by Lipofectamine LTX (Invitrogen); for PC12 cells and cardiac myocytes, the glasses were coated with poly-D-lysine and plus reagent (Invitrogen) was added for the transfection. Primary cultures of the cardiac myocytes were isolated from 1-day-old Crlj: CD (SD) rats (Charles River).

Before imaging, culture medium was replaced by Hanks' balanced salt solution (HBSS) for HeLa cells and cardiac myocytes or Krebs-Ringer-HEPES (KRH) buffer [24] for PC12 cells. Cells were imaged on an inverted microscope (TE300, Nikon) with a xenon lamp (C6979, Hamamatsu), a 100× oil immersion objective lens (S Fluor, Nikon), a quad channel imager (Quad-View, Optical Insights), and a cooled CCD camera (CoolSNAP HQ, Roper scientific), and were maintained at 37°C during all imaging experiments. The quad channel imager mounted three dichroic mirrors: Q500LP; Q530LP; Q570LP, and four emission filters: HQ487/25m; HQ515/30m; HQ550/40m; HQ590/40m. Excitation filters HQ405/20× or D380/10× were used for dual FRET experiments. For comparison of red cameleons, images of directly excited Sapphire and RFP were acquired with a 10× objective lens (Plan Fluor, Nikon) and excitation filters HQ405/20× and S550/20× and emission filters S535/30m and S605/40m without the quad channel imager, respectively. For all excitations, we commonly used a dichroic mirror 455DCLP in the microscope modified as previously described [34]. All filters were obtained from Chroma Technology. Whole system was controlled by using MetaMorph software (Universal Imaging). Timelapse intervals were 50–100 ms and 2.5 s for cardiac myocytes and PC12 cells, respectively, and exposure time was 50–100 ms (4×4 or 8×8 binning). Excitation light is attenuated by a 25% transmittance neutral density (ND) filter to reduce photobleaching, as necessary.

### Image analysis

For geometrical distortion and uneven intensity distribution of the acquired images by using a quad channel imager (see Fig. S1D), we applied two corrections to the images as preprocessing of linear unmixing. First, we minimized the geometrical distortion using projective transformation [20]. In planer projective transformation, a transform has the following form:
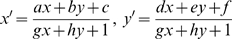
where *x* and *y* are the coordinates of a point before the transformation, and *x′* and *y′* are the coordinates of the corresponding point after, and *a–h* are the transformation coefficients. To obtain eight equations for the *a–h*, we assigned the coordinates of each vertex of the detection channels (see Fig. S1A) to *x* and *y*, and the average of *x* and *y* for all channels to *x′* and *y′*. Then we solved their simultaneous equations, and calculated the (*x′*, *y′*) of any points in the acquired images using the found *a–h*. Intensity at (*x′*, *y′*) in the corrected images was calculated approximately from the acquired images by bilinear interpolation [35].

After this correction, uneven distribution of fluorescence intensity in each channel still remained (see Fig. S1E); depending on the pixel in each channel, fluorescence leakages were different. Thus, we second normalized the distribution using images of a dye mixture (see Fig. S1B). Solution containing 0.5 mM fura-2 AM, 0.5 mM Calcium Orange AM (Invitrogen) and 2.5 mM CaCl_2_ was dropped on the glass-bottom dish, and the fluorescence images for four-color channels were acquired. Cell images were normalized by this reference image of each channel to correct uneven intensity distribution (see Fig. S1F).

Upon the corrections as described above, we acquired the fluorescence leakages of four fluorescent proteins expressed in HeLa cells into each channel. And then we applied linear unmixing as previously described [8]. In each pixel, the relation between the unknown contribution of each fluorescent protein *x* and the mixed (acquired) fluorescence signals in each detection channel *y* could be represented as:
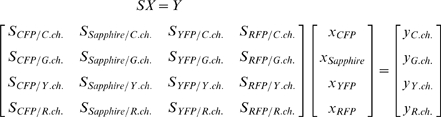
where *S* is a spectral matrix composed with the reference of individual fluorescent proteins. *X* was obtained by multiplying the inverse of *S* on the left of *Y*.

Images were smoothed with a 3×3 median filter to reduce noise and subtracted background before the image processing. Programs for two corrections and linear unmixing were written in ANSI C and built with gcc in Cygwin (Red Hat). The other image processing and representing pseudocolor ratio images in intensity-modulated display mode were done by MetaMorph software.

To measure Ca^2+^ oscillation frequency of cardiac myocytes, a series of eleven spikes was picked up from a trace of SapRC2.12, and an average of ten intervals between the neighboring peaks was calculated. An increase rate of frequency was calculated as the ratio of after to before isoproterenol application in the same cell.

## Supporting Information

Figure S1 Correction of geometrical distortion and uneven intensity distribution caused by a quad channel imager. (A) Edges of C. (cyan), G. (green), Y. (yellow), and R. ch. (red) of an acquired image. Enlarged view of boxed area in upper is shown (lower). A sobel filter was used for edge detection. Scale bar, 5 µm. (B) Pseudocolor images of fluorescence intensity of a dye mixture in C., G., Y., and R. ch., indicating the uneven intensity distribution. Scale bar, 10 µm. (C–F) Representative images of a HeLa cell expressing T-Sapphire. The acquired image in G. ch. (C), its ratio image of Y. ch. to G. ch. intensities with no correction (D), with only correction for the geometrical distortion (E), and with subsequent correction of the uneven intensity distribution using reference of the dye mixture (F). Scale bar, 10 µm.
(0.65 MB TIF)

Figure S2 cAMP imaging by using Epac1-camps in PC12 cells. Typical response to stimulation with 5 Î¼M adenosine and 100 Î¼M IBMX for cAMP and subsequent stimulation with 2 Î¼M SNAP for cGMP is shown (*n* = 11). Scale bar, 10 Î¼m.
(0.08 MB TIF)

Figure S3 Artifact without linear unmixing in simultaneous imaging of cAMP and cGMP within the PC12 cell. Traces in the cell shown in Figure 3 are represented.
(0.15 MB TIF)

Figure S4 cAMP imaging by using Epac1-camps in spontaneously contracting cardiac myocytes. Typical response to stimulation with 10 µM isoproterenol is shown (*n* = 5). Scale bar, 10 µm.
(0.14 MB TIF)

Figure S5 Artifact without linear unmixing in simultaneous imaging of cAMP and Ca^2+^ within the cardiac myocyte. Traces in the cell shown in Figure 5 are represented. Arrowhead indicates the artifact caused by contraction of the cell.
(0.24 MB TIF)
