# Supplementary figures and images for: Simultaneous Live Cell Imaging Using Dual FRET Sensors with a Single Excitation Light
Source: PLoS One. 2009 Jun 24;4(6):e6036. doi: 10.1371/journal.pone.0006036 (PMC2696040; doi:10.1371/journal.pone.0006036)

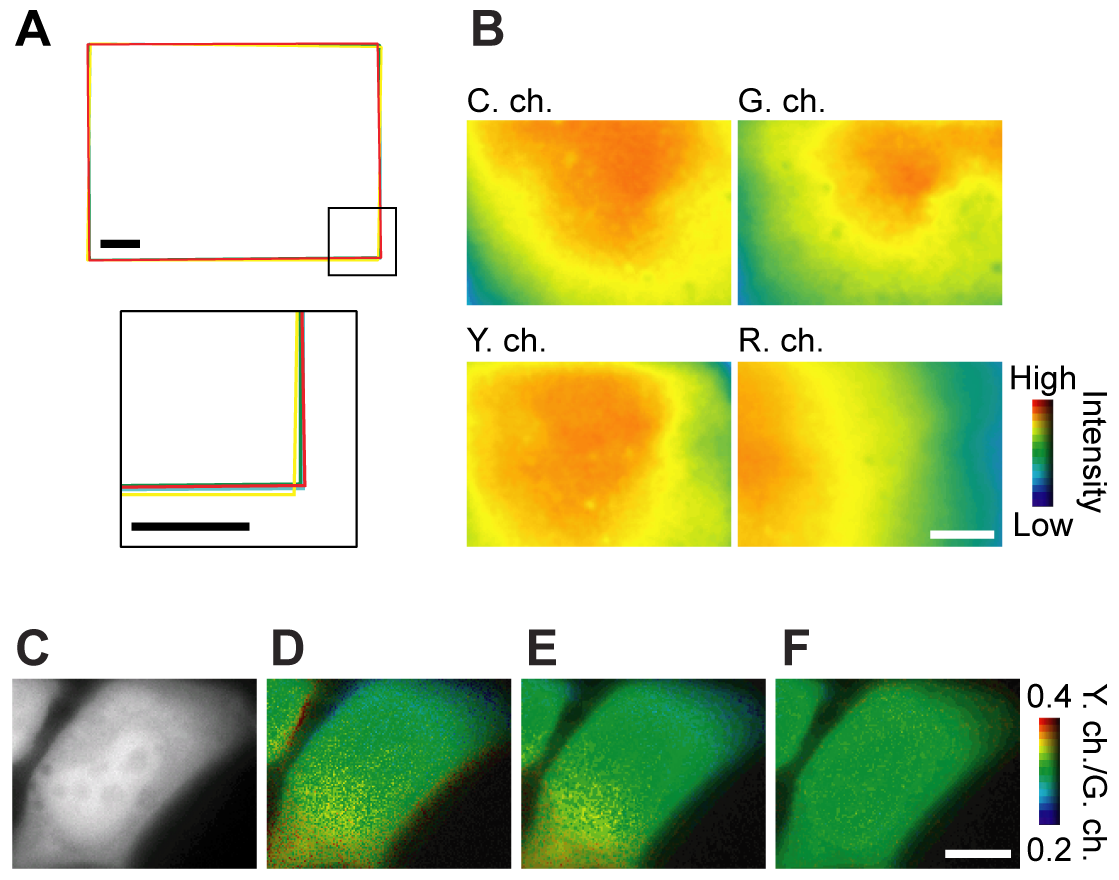

Supplement: Figure S1 — Correction of geometrical distortion and uneven intensity distribution caused by a quad channel imager. (A) Edges of C. (cyan), G. (green), Y. (yellow), and R. ch. (red) of an acquired image. Enlarged view of boxed area in upper is shown (lower). A sobel filter was used for edge detection. Scale bar, 5 µm. (B) Pseudocolor images of fluorescence intensity of a dye mixture in C., G., Y., and R. ch., indicating the uneven intensity distribution. Scale bar, 10 µm. (C–F) Representative images of a HeLa cell expressing T-Sapphire. The acquired image in G. ch. (C), its ratio image of Y. ch. to G. ch. intensities with no correction (D), with only correction for the geometrical distortion (E), and with subsequent correction of the uneven intensity distribution using reference of the dye mixture (F). Scale bar, 10 µm. (0.65 MB TIF) [file pone.0006036.s001.tif]

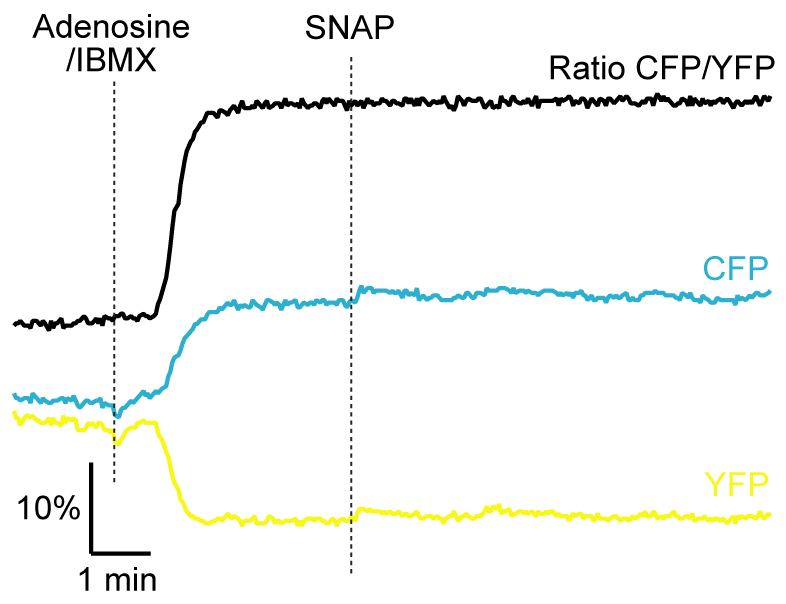

Supplement: Figure S2 — cAMP imaging by using Epac1-camps in PC12 cells. Typical response to stimulation with 5 Î¼M adenosine and 100 Î¼M IBMX for cAMP and subsequent stimulation with 2 Î¼M SNAP for cGMP is shown (n = 11). Scale bar, 10 Î¼m. (0.08 MB TIF) [file pone.0006036.s002.tif]

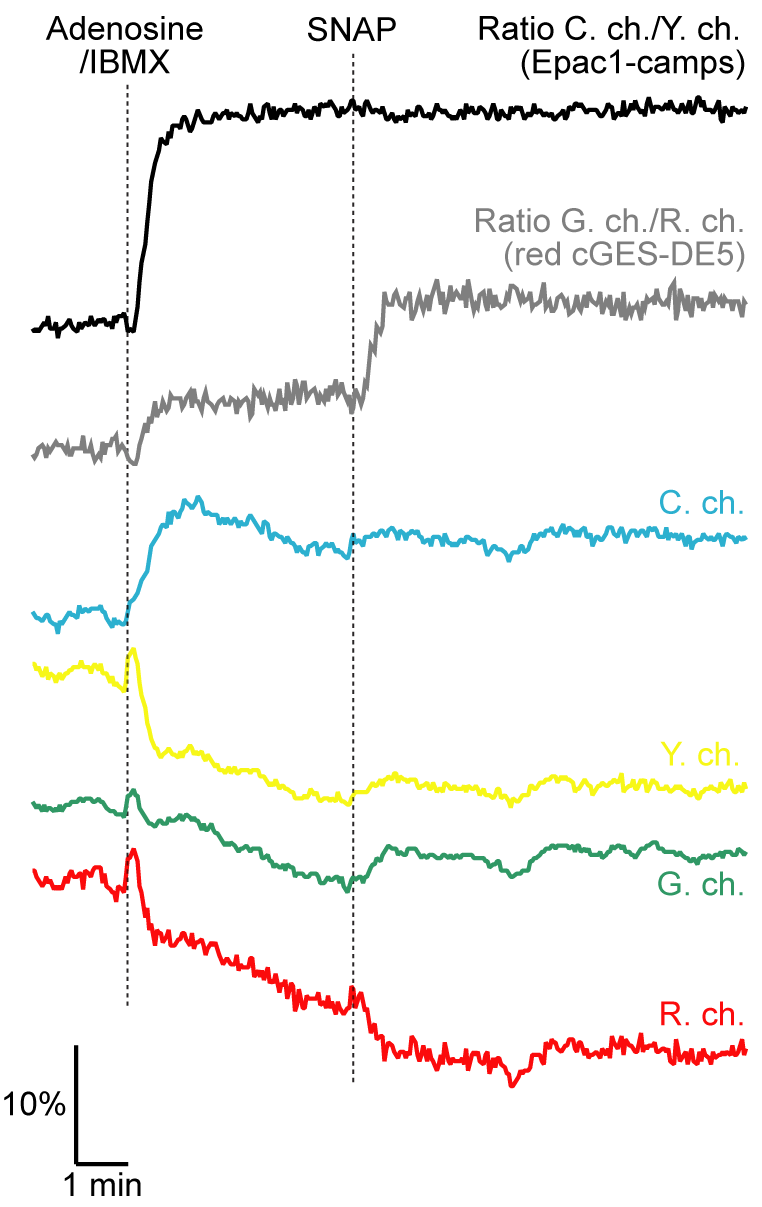

Supplement: Figure S3 — Artifact without linear unmixing in simultaneous imaging of cAMP and cGMP within the PC12 cell. Traces in the cell shown in Figure 3 are represented. (0.15 MB TIF) [file pone.0006036.s003.tif]

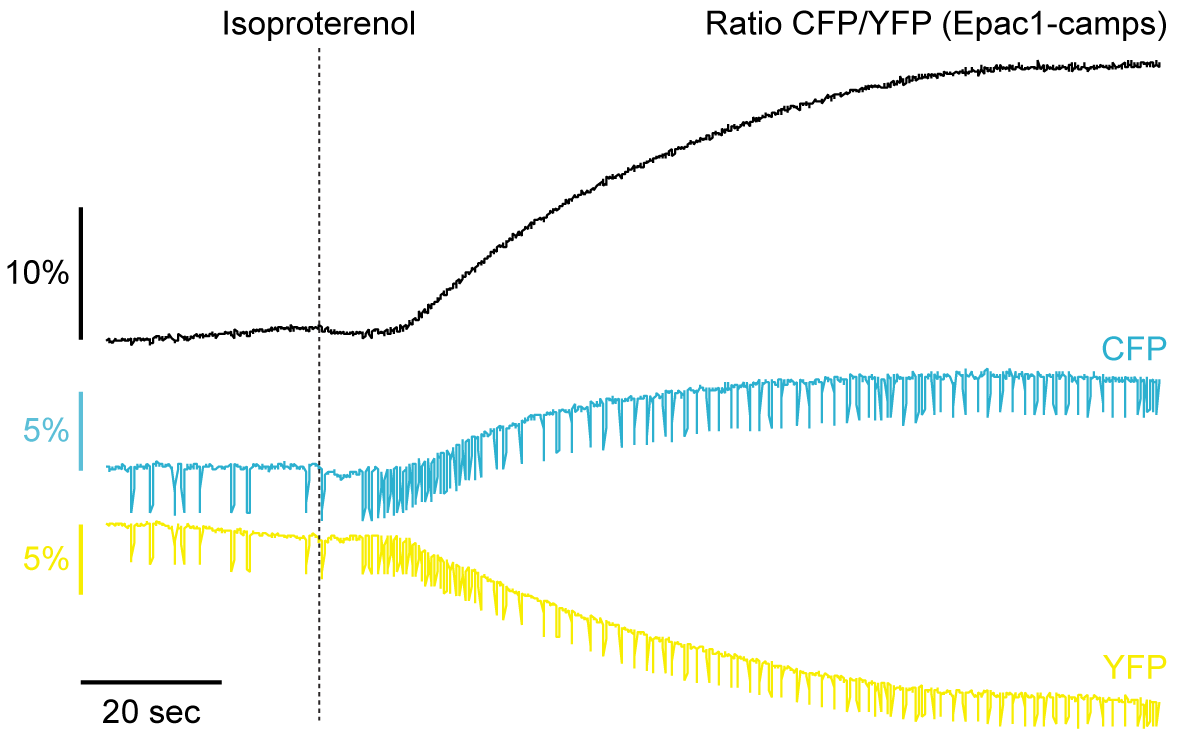

Supplement: Figure S4 — cAMP imaging by using Epac1-camps in spontaneously contracting cardiac myocytes. Typical response to stimulation with 10 µM isoproterenol is shown (n = 5). Scale bar, 10 µm. (0.14 MB TIF) [file pone.0006036.s004.tif]

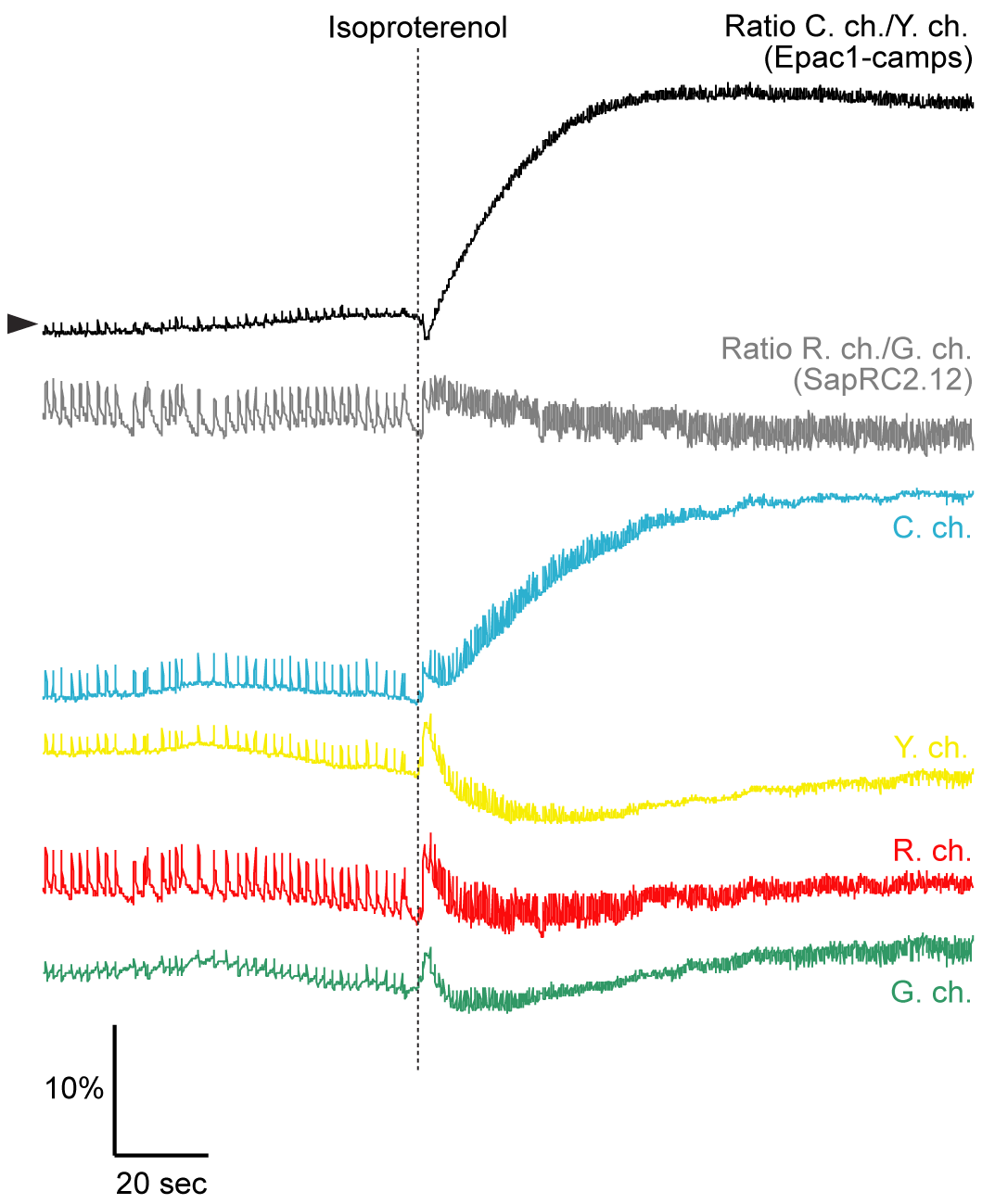

Supplement: Figure S5 — Artifact without linear unmixing in simultaneous imaging of cAMP and Ca2+ within the cardiac myocyte. Traces in the cell shown in Figure 5 are represented. Arrowhead indicates the artifact caused by contraction of the cell. (0.24 MB TIF) [file pone.0006036.s005.tif]
